# Poly[diaquabis­(μ_4_-fumarato-κ^4^ *O* ^1^:*O* ^1′^:*O* ^4^:*O* ^4′^)(μ_4_-fumarato-κ^6^ *O* ^1^:*O* ^1^,*O* ^1′^:*O* ^4^:*O* ^4^,*O* ^4′^)(μ_2_-fumaric acid-κ^2^ *O* ^1^:*O* ^4^)dipraseodymium(III)]

**DOI:** 10.1107/S1600536811038347

**Published:** 2011-09-30

**Authors:** Pei-lian Liu, Wanwan Cao, Jin Wang, Rong-hua Zeng, Zhuo Zeng

**Affiliations:** a School of Chemistry and Environment, South China Normal University, Guangzhou 510006, People’s Republic of China; b Key Laboratory of Organofluorine Chemistry, Shanghai Institute of Organic Chemistry, Chinese Academy of Sciences, Shanghai, 200032, People’s Republic of China

## Abstract

The title complex, [Pr_2_(C_4_H_2_O_4_)_3_(C_4_H_4_O_4_)(H_2_O)_2_]_*n*_, was synthesized by reaction of praseodymium(III) nitrate hexa­hydrate with fumaric acid in a water–ethanol (4:1) solution. The asymmetric unit comprises a Pr^3+^ cation, one and a half fumarate dianions (*L* ^2−^), one half-mol­ecule of fumaric acid (*H_2_L*) and one coordinated water mol­ecule. The carboxyl­ate groups of the fumarate dianion and fumaric acid exhibit different coordination modes. In one fumarate dianion, two carboxyl­ate groups are chelating with two Pr^3+^ cations, and the other two O atoms each coordinate a Pr^3+^ cation. Each O atom of the second fumarate dianion binds to a different Pr^3+^ cation. The fumaric acid employs one O atom at each end to bridge two Pr^3+^ cations. The Pr^3+^ cation is coordinated in a distorted tricapped trigonal–prismatic environment by eight O atoms of fumarate dianion or fumaric acid ligands and one water O atom. The PrO_9_ coordination polyhedra are edge-shared through one carboxyl­ate O atom and two carboxyl­ate groups, generating infinite praseodymium–oxygen chains, which are further connected by the ligands into a three-dimensional framework. The crystal structure is stabilized by O—H⋯O hydrogen-bond inter­actions between the coordin­ated water mol­ecule and the carboxyl­ate O atoms.

## Related literature

For the structural diversity and potential use as superconductors and magnetic materials of metal complexes of carboxyl­ates, see: Kim *et al.* (2004 ▶); Ye *et al.* (2005 ▶). For applications of rare earth carboxyl­ates, see: Baggio & Perec (2004 ▶); Seo *et al.* (2000 ▶).
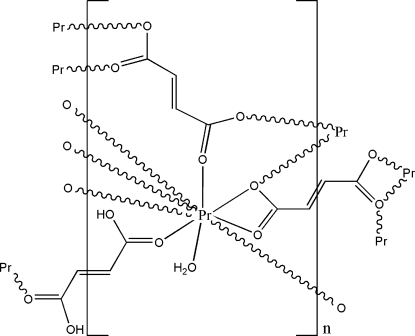


## Experimental

### 

#### Crystal data

[Pr_2_(C_4_H_2_O_4_)_3_(C_4_H_4_O_4_)(H_2_O)_2]_]
*M* *_r_* = 776.10
Monoclinic, 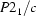

*a* = 8.3714 (3) Å
*b* = 14.6034 (6) Å
*c* = 8.7518 (4) Å
β = 103.118 (2)°
*V* = 1042.00 (7) Å^3^
*Z* = 2
Mo *K*α radiation
μ = 4.72 mm^−1^
*T* = 298 K
0.26 × 0.19 × 0.15 mm

#### Data collection

Bruker APEXII CCD area-detector diffractometer
Absorption correction: multi-scan (*SADABS*; Sheldrick, 1996 ▶) *T*_min_ = 0.355, *T*_max_ = 0.493
10102 measured reflections
2394 independent reflections
2175 reflections with *I* > 2σ(*I*)
*R*_int_ = 0.027

#### Refinement

*R*[*F* ^2^ > 2σ(*F* ^2^)] = 0.016
*wR*(*F* ^2^) = 0.041
*S* = 1.05
2394 reflections
170 parameters
3 restraints
H atoms treated by a mixture of independent and constrained refinement
Δρ_max_ = 0.44 e Å^−3^
Δρ_min_ = −0.75 e Å^−3^

### 

Data collection: *APEX2* (Bruker, 2008 ▶); cell refinement: *SAINT* (Bruker, 2008 ▶); data reduction: *SAINT*; program(s) used to solve structure: *SHELXS97* (Sheldrick, 2008 ▶); program(s) used to refine structure: *SHELXL97* (Sheldrick, 2008 ▶); molecular graphics: *SHELXTL* (Sheldrick, 2008 ▶); software used to prepare material for publication: *SHELXL97*.

## Supplementary Material

Crystal structure: contains datablock(s) global, I. DOI: 10.1107/S1600536811038347/hg5089sup1.cif

Structure factors: contains datablock(s) I. DOI: 10.1107/S1600536811038347/hg5089Isup2.hkl

Additional supplementary materials:  crystallographic information; 3D view; checkCIF report

## Figures and Tables

**Table 1:** Hydrogen-bond geometry (Å, °)

*D*—H⋯*A*	*D*—H	H⋯*A*	*D*⋯*A*	*D*—H⋯*A*
C3—H3⋯O7	0.93	2.50	2.815 (3)	100
O1*W*—H2*W*⋯O7^i^	0.83 (1)	2.11 (1)	2.911 (2)	165 (2)
O2—H2*A*⋯O9^ii^	0.82	1.86	2.661 (2)	167
O1*W*—H1*W*⋯O5^iii^	0.82 (1)	2.09 (2)	2.816 (2)	147 (2)

Symmetry codes: (i) 
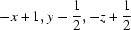
; (ii) 
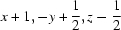
; (iii) 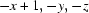.

**Fig. 1.**
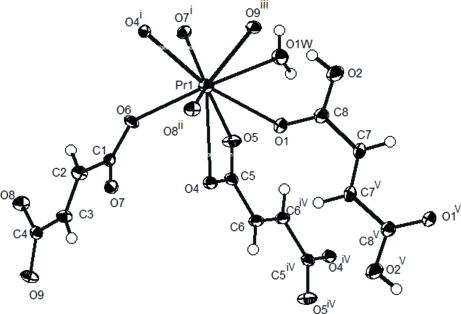
View of the local coordination of praseodymium(III) with the atom-numbering scheme. Displacement ellipsoids are drawn at the 50% probability level. [Symmetry codes: (i)x,1/2 - y,-1/2 + z; (ii)1 + x,y,z; (iii)1 + x,1/2 - y,-1/2 + z; (iv)1 - x,-y,1 - z; (v)2 - x,-y,1 - z.]

**Fig. 2.**
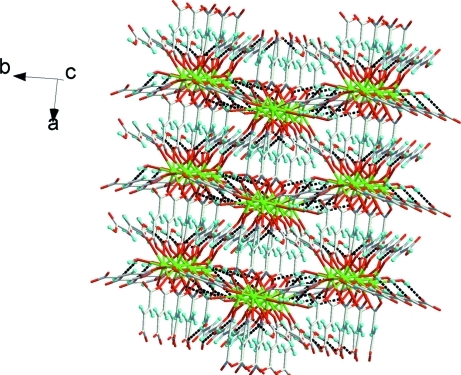
Perspective view of the crystal packing.

## supplementary crystallographic information

### Comment

Metal complexes of carboxylates have attracted much attention due to their wide range of structural diversities and potential use on superconductors and magnetic materials (Kim *et al.*, 2004; Ye *et al.*, 2005). What is more, a particularly attractive goal is the rare-earth carboxylates, because of their special application on the 4f-block elements and their unique f-f electronic transitions. (Seo *et al.*, 2000; Baggio *et al.*, 2004). In this paper, we report the title complex (scheme. 1), obtained by the reaction of praseodymium(III) nitrate hexahydrate with fumaric acid in a water-ethanol (4:1) solution.

The structure of the asymmetric unit of the title complex is shown in Fig. 1. It comprises a Pr^3+^ cation, 1.5 fumarate dianions (*L*^2-^), 0.5 fumaric acid (*H_2_L*) and one water ligand. The carboxylate groups of the fumarate dianion and fumaric acid exhibit different coordination modes. In one fumarate dianion, two carboxylate groups are chelating with two Pr^3+^ cations, and other two O atoms(O4 and O4^iv^) are coordinated with Pr^3+^ cation respectively. The other fumarate dianion bridges four Pr^3+^ cations with monodentate mode, and the fumaric acid bridges two Pr^3+^ cations with monodentate mode. In the crystallographic asymmetric unit, the Pr^3+^ cation is sited within a distorted tricapped trigonal prism defined by nine O atoms derived from seven different bridging ligands and a coordinated water molecule. One of the carboxylate groups, derived from *L*^2-^, is chelating, and the remaining six carboxylates coordinate in a monodentate mode. The Pr—O bond distances range from 2.4040 (15) to 2.7719 (16) Å. The O—Pr—O bond angles range from 72.35 (5) to 146.04 (5)°. The PrO_9_ coordination polyhedra are edge-shared through one carboxylate O atoms (O4) and two carboxylate groups (O8—C4—O9 and O6—C1—O7) to generate infinite praseodymium-oxygen chains (Fig. 2). The chains are further connected by the ligands to form a three-dimensional framework. The crystal is stabilized by hydrogen bond interactions between the coordinated water and carboxylate O atoms.

### Experimental

Fumaric acid (0.5 mmol, 0.058 g), Praseodymium(III) nitrate hexahydrate(0.3 mmol, 0.13 g) was dissolved in a water-ethanol(4:1) solution(10 ml). The mixture was transferred to a 20 ml Teflon-lined stainless steel autoclave, which was heated at 413 K for 96 h. The reactor was cooled to room temperature over a period of 24 h. Blue crystals were obtained after filtrated, washed with water and vacuum dried.

### Refinement

Carbon-bound H atoms were included in the riding-model approximation, with C—H =0.93Å and with *U*_iso_(H) = 1.2*U*_eq_(C). H atom bound to carboxyl-O atom was initially located in a difference map but was then fixed in the riding-model approximation, with O—H = 0.82Å and with *U*_iso_(H) = 1.5 *U*_eq_(O). Water H atoms were tentatively located in difference Fourier maps and were refined with distance restraints of O—H = 0.82Å and H···H = 1.29 Å, and with *U*_iso_(H) = 1.5 *U*_eq_(O).

### Crystal data

**Table:**

[Pr_2_(C_4_H_2_O_4_)_3_(C_4_H_4_O_4_)(H_2_O)_2_]	*F*(000) = 744
*M**_r_* = 776.10	*D*_x_ = 2.474 Mg m^−^^3^
Monoclinic, *P*2_1_/*c*	Mo *K*α radiation, λ = 0.71073 Å
Hall symbol: -P 2ybc	Cell parameters from 7002 reflections
*a* = 8.3714 (3) Å	θ = 2.5–27.5°
*b* = 14.6034 (6) Å	µ = 4.72 mm^−^^1^
*c* = 8.7518 (4) Å	*T* = 298 K
β = 103.118 (2)°	Block, blue
*V* = 1042.00 (7) Å^3^	0.26 × 0.19 × 0.15 mm
*Z* = 2	

### Data collection

**Table:**

Bruker APEXII CCD area-detector diffractometer	2394 independent reflections
Radiation source: fine-focus sealed tube	2175 reflections with *I* > 2σ(*I*)
graphite	*R*_int_ = 0.027
phi and ω scans	θ_max_ = 27.5°, θ_min_ = 2.5°
Absorption correction: multi-scan (*SADABS*; Sheldrick, 1996)	*h* = −9→10
*T*_min_ = 0.355, *T*_max_ = 0.493	*k* = −16→18
10102 measured reflections	*l* = −11→6

### Refinement

**Table:**

Refinement on *F*^2^	Primary atom site location: structure-invariant direct methods
Least-squares matrix: full	Secondary atom site location: difference Fourier map
*R*[*F*^2^ > 2σ(*F*^2^)] = 0.016	Hydrogen site location: inferred from neighbouring sites
*wR*(*F*^2^) = 0.041	H atoms treated by a mixture of independent and constrained refinement
*S* = 1.05	*w* = 1/[σ^2^(*F*_o_^2^) + (0.0212*P*)^2^ + 0.5013*P*] where *P* = (*F*_o_^2^ + 2*F*_c_^2^)/3
2394 reflections	(Δ/σ)_max_ = 0.001
170 parameters	Δρ_max_ = 0.44 e Å^−^^3^
3 restraints	Δρ_min_ = −0.75 e Å^−^^3^

### Special details

**Table:**

Geometry. All e.s.d.'s (except the e.s.d. in the dihedral angle between two l.s. planes) are estimated using the full covariance matrix. The cell e.s.d.'s are taken into account individually in the estimation of e.s.d.'s in distances, angles and torsion angles; correlations between e.s.d.'s in cell parameters are only used when they are defined by crystal symmetry. An approximate (isotropic) treatment of cell e.s.d.'s is used for estimating e.s.d.'s involving l.s. planes.
Refinement. Refinement of *F*^2^ against ALL reflections. The weighted *R*-factor *wR* and goodness of fit *S* are based on *F*^2^, conventional *R*-factors *R* are based on *F*, with *F* set to zero for negative *F*^2^. The threshold expression of *F*^2^ > σ(*F*^2^) is used only for calculating *R*-factors(gt) *etc*. and is not relevant to the choice of reflections for refinement. *R*-factors based on *F*^2^ are statistically about twice as large as those based on *F*, and *R*- factors based on ALL data will be even larger.

### Fractional atomic coordinates and isotropic or equivalent isotropic displacement parameters (Å^2^)

**Table:**

	*x*	*y*	*z*	*U*_iso_*/*U*_eq_	
C1	0.3406 (3)	0.31371 (13)	0.2443 (3)	0.0132 (5)	
C2	0.1598 (3)	0.31134 (14)	0.2330 (3)	0.0170 (5)	
H2	0.0902	0.2974	0.1369	0.020*	
C3	0.0955 (3)	0.32820 (15)	0.3540 (3)	0.0155 (4)	
H3	0.1665	0.3451	0.4476	0.019*	
C4	−0.0831 (3)	0.32223 (14)	0.3522 (3)	0.0128 (4)	
C5	0.5360 (3)	0.10365 (14)	0.3762 (2)	0.0133 (4)	
C6	0.5244 (3)	0.04325 (14)	0.5105 (3)	0.0160 (4)	
H6	0.5522	0.0668	0.6118	0.019*	
C8	0.9749 (3)	0.06063 (16)	0.3044 (3)	0.0191 (5)	
O1	0.8615 (2)	0.11349 (12)	0.30016 (19)	0.0244 (4)	
O2	1.0535 (2)	0.05332 (15)	0.1918 (2)	0.0356 (5)	
H2A	1.0106	0.0868	0.1187	0.053*	
C7	1.0333 (3)	−0.00121 (16)	0.4388 (3)	0.0199 (5)	
H7	1.1178	−0.0422	0.4369	0.024*	
O4	0.5972 (2)	0.18329 (9)	0.40478 (19)	0.0143 (3)	
O5	0.4920 (2)	0.07499 (11)	0.23774 (17)	0.0208 (4)	
O6	0.39185 (18)	0.27539 (11)	0.13689 (17)	0.0174 (3)	
O7	0.43025 (18)	0.35433 (10)	0.36074 (17)	0.0149 (3)	
O8	−0.1853 (2)	0.30866 (10)	0.2257 (2)	0.0174 (4)	
O9	−0.1193 (2)	0.33071 (11)	0.48376 (19)	0.0182 (3)	
O1W	0.6829 (2)	0.02369 (11)	0.02361 (19)	0.0198 (4)	
H2W	0.645 (3)	−0.0181 (14)	0.068 (2)	0.030*	
H1W	0.656 (3)	0.0080 (17)	−0.0692 (12)	0.030*	
Pr1	0.632685 (13)	0.190489 (7)	0.097718 (13)	0.00921 (5)	

### Atomic displacement parameters (Å^2^)

**Table:**

	*U*^11^	*U*^22^	*U*^33^	*U*^12^	*U*^13^	*U*^23^
C1	0.0123 (11)	0.0141 (11)	0.0138 (12)	0.0023 (7)	0.0040 (9)	0.0036 (8)
C2	0.0118 (11)	0.0233 (13)	0.0153 (13)	−0.0014 (8)	0.0018 (10)	−0.0034 (8)
C3	0.0116 (10)	0.0221 (11)	0.0132 (11)	−0.0022 (8)	0.0035 (9)	−0.0013 (9)
C4	0.0139 (11)	0.0108 (10)	0.0139 (11)	−0.0008 (8)	0.0039 (9)	−0.0009 (8)
C5	0.0156 (10)	0.0123 (10)	0.0139 (11)	−0.0005 (8)	0.0071 (9)	−0.0005 (8)
C6	0.0226 (12)	0.0147 (11)	0.0117 (11)	−0.0013 (9)	0.0063 (9)	0.0005 (8)
C8	0.0173 (11)	0.0225 (12)	0.0171 (12)	0.0025 (9)	0.0031 (10)	0.0016 (9)
O1	0.0270 (9)	0.0292 (10)	0.0171 (9)	0.0133 (7)	0.0055 (8)	0.0058 (7)
O2	0.0323 (11)	0.0532 (13)	0.0258 (10)	0.0204 (9)	0.0160 (9)	0.0184 (9)
C7	0.0188 (11)	0.0219 (12)	0.0174 (12)	0.0080 (9)	0.0008 (9)	0.0023 (9)
O4	0.0200 (8)	0.0108 (7)	0.0129 (8)	−0.0021 (6)	0.0054 (7)	−0.0012 (6)
O5	0.0348 (10)	0.0173 (8)	0.0117 (8)	−0.0093 (7)	0.0082 (7)	−0.0021 (6)
O6	0.0152 (8)	0.0242 (8)	0.0144 (8)	0.0039 (6)	0.0068 (7)	−0.0032 (6)
O7	0.0135 (7)	0.0168 (8)	0.0136 (8)	0.0011 (6)	0.0013 (6)	−0.0012 (6)
O8	0.0142 (8)	0.0217 (9)	0.0152 (9)	−0.0037 (6)	0.0013 (7)	−0.0020 (6)
O9	0.0172 (8)	0.0240 (8)	0.0159 (9)	−0.0035 (7)	0.0089 (7)	−0.0041 (7)
O1W	0.0248 (9)	0.0150 (8)	0.0201 (9)	−0.0034 (6)	0.0057 (8)	−0.0025 (7)
Pr1	0.00954 (7)	0.01013 (7)	0.00840 (8)	0.00028 (4)	0.00300 (5)	0.00065 (4)

### Geometric parameters (Å, °)

**Table:**

C1—O6	1.251 (3)	O1—Pr1	2.5560 (16)
C1—O7	1.268 (3)	O2—H2A	0.8200
C1—O7	1.268 (3)	C7—C7^ii^	1.316 (5)
C1—C2	1.495 (3)	C7—H7	0.9300
C2—C3	1.315 (3)	O4—Pr1^iii^	2.4717 (14)
C2—H2	0.9300	O4—Pr1	2.7719 (16)
C3—C4	1.495 (3)	O5—Pr1	2.5278 (15)
C3—H3	0.9300	O6—Pr1	2.4563 (14)
C4—O8	1.252 (3)	O7—Pr1^iii^	2.4508 (15)
C4—O9	1.261 (3)	O8—Pr1^iv^	2.4040 (15)
C5—O5	1.256 (2)	O9—Pr1^v^	2.5184 (15)
C5—O4	1.273 (2)	O1W—Pr1	2.5795 (16)
C5—C6	1.490 (3)	O1W—H2W	0.825 (10)
C6—C6^i^	1.328 (4)	O1W—H1W	0.824 (9)
C6—H6	0.9300	Pr1—O8^vi^	2.4040 (15)
C8—O1	1.218 (3)	Pr1—O7^vii^	2.4508 (15)
C8—O2	1.308 (3)	Pr1—O4^vii^	2.4717 (14)
C8—C7	1.475 (3)	Pr1—O9^viii^	2.5184 (15)
			
O6—C1—O7	124.9 (2)	H2W—O1W—H1W	102.5 (14)
O6—C1—O7	124.9 (2)	O8^vi^—Pr1—O7^vii^	146.04 (5)
O6—C1—C2	117.1 (2)	O8^vi^—Pr1—O6	91.49 (5)
O7—C1—C2	118.04 (19)	O7^vii^—Pr1—O6	79.69 (5)
O7—C1—C2	118.04 (19)	O8^vi^—Pr1—O4^vii^	75.42 (5)
C3—C2—C1	122.4 (2)	O7^vii^—Pr1—O4^vii^	70.62 (5)
C3—C2—H2	118.8	O6—Pr1—O4^vii^	75.15 (5)
C1—C2—H2	118.8	O8^vi^—Pr1—O9^viii^	77.32 (5)
C2—C3—C4	124.9 (2)	O7^vii^—Pr1—O9^viii^	96.08 (5)
C2—C3—H3	117.6	O6—Pr1—O9^viii^	153.38 (5)
C4—C3—H3	117.6	O4^vii^—Pr1—O9^viii^	78.65 (5)
O8—C4—O9	124.4 (2)	O8^vi^—Pr1—O5	124.67 (5)
O8—C4—C3	120.0 (2)	O7^vii^—Pr1—O5	85.60 (5)
O9—C4—C3	115.6 (2)	O6—Pr1—O5	77.38 (6)
O5—C5—O4	120.73 (19)	O4^vii^—Pr1—O5	146.29 (5)
O5—C5—C6	120.43 (19)	O9^viii^—Pr1—O5	128.82 (5)
O4—C5—C6	118.78 (19)	O8^vi^—Pr1—O1	72.35 (5)
C6^i^—C6—C5	121.8 (3)	O7^vii^—Pr1—O1	137.28 (5)
C6^i^—C6—H6	119.1	O6—Pr1—O1	129.43 (5)
C5—C6—H6	119.1	O4^vii^—Pr1—O1	139.16 (5)
O1—C8—O2	123.4 (2)	O9^viii^—Pr1—O1	70.41 (5)
O1—C8—C7	121.8 (2)	O5—Pr1—O1	74.27 (5)
O2—C8—C7	114.8 (2)	O8^vi^—Pr1—O1W	132.29 (5)
C8—O1—Pr1	139.18 (16)	O7^vii^—Pr1—O1W	69.87 (5)
C8—O2—H2A	109.5	O6—Pr1—O1W	134.22 (5)
C7^ii^—C7—C8	120.5 (3)	O4^vii^—Pr1—O1W	122.25 (5)
C7^ii^—C7—H7	119.7	O9^viii^—Pr1—O1W	65.70 (5)
C8—C7—H7	119.7	O5—Pr1—O1W	67.18 (5)
C5—O4—Pr1^iii^	142.77 (14)	O1—Pr1—O1W	67.64 (5)
C5—O4—Pr1	88.35 (12)	O8^vi^—Pr1—O4	76.79 (5)
Pr1^iii^—O4—Pr1	127.68 (5)	O7^vii^—Pr1—O4	127.21 (5)
C5—O5—Pr1	100.28 (12)	O6—Pr1—O4	67.16 (5)
C1—O6—Pr1	139.76 (15)	O4^vii^—Pr1—O4	131.91 (3)
C1—O7—Pr1^iii^	136.47 (13)	O9^viii^—Pr1—O4	131.20 (5)
C4—O8—Pr1^iv^	139.42 (14)	O5—Pr1—O4	48.73 (4)
C4—O9—Pr1^v^	137.70 (14)	O1—Pr1—O4	62.59 (5)
Pr1—O1W—H2W	118.5 (18)	O1W—Pr1—O4	105.50 (5)
Pr1—O1W—H1W	119.2 (19)		
			
O6—C1—C2—C3	162.7 (2)	C1—O6—Pr1—O1	16.7 (3)
O7—C1—C2—C3	−17.5 (3)	C1—O6—Pr1—O1W	113.2 (2)
O7—C1—C2—C3	−17.5 (3)	C1—O6—Pr1—O4	23.5 (2)
C1—C2—C3—C4	−176.7 (2)	C5—O5—Pr1—O8^vi^	4.60 (16)
C2—C3—C4—O8	−7.0 (3)	C5—O5—Pr1—O7^vii^	−158.77 (14)
C2—C3—C4—O9	172.2 (2)	C5—O5—Pr1—O6	−78.37 (13)
O5—C5—C6—C6^i^	3.3 (4)	C5—O5—Pr1—O4^vii^	−114.41 (14)
O4—C5—C6—C6^i^	−173.9 (3)	C5—O5—Pr1—O9^viii^	106.95 (14)
O2—C8—O1—Pr1	29.0 (4)	C5—O5—Pr1—O1	59.30 (13)
C7—C8—O1—Pr1	−150.46 (18)	C5—O5—Pr1—O1W	131.23 (14)
O1—C8—C7—C7^ii^	−2.2 (5)	C5—O5—Pr1—O4	−7.80 (12)
O2—C8—C7—C7^ii^	178.3 (3)	C8—O1—Pr1—O8^vi^	−115.0 (3)
O5—C5—O4—Pr1^iii^	153.25 (17)	C8—O1—Pr1—O7^vii^	44.8 (3)
C6—C5—O4—Pr1^iii^	−29.5 (3)	C8—O1—Pr1—O6	168.1 (2)
O5—C5—O4—Pr1	−13.4 (2)	C8—O1—Pr1—O4^vii^	−75.5 (3)
C6—C5—O4—Pr1	163.80 (18)	C8—O1—Pr1—O9^viii^	−32.5 (2)
O4—C5—O5—Pr1	15.0 (2)	C8—O1—Pr1—O5	109.8 (3)
C6—C5—O5—Pr1	−162.19 (17)	C8—O1—Pr1—O1W	38.5 (2)
O7—C1—O6—Pr1	33.2 (3)	C8—O1—Pr1—O4	161.1 (3)
O7—C1—O6—Pr1	33.2 (3)	C5—O4—Pr1—O8^vi^	−161.98 (13)
C2—C1—O6—Pr1	−147.04 (17)	Pr1^iii^—O4—Pr1—O8^vi^	28.18 (8)
O6—C1—O7—O7	0.00 (13)	C5—O4—Pr1—O7^vii^	44.97 (14)
C2—C1—O7—O7	0.00 (7)	Pr1^iii^—O4—Pr1—O7^vii^	−124.87 (8)
O6—C1—O7—Pr1^iii^	−70.6 (3)	C5—O4—Pr1—O6	100.68 (13)
O7—C1—O7—Pr1^iii^	0(100)	Pr1^iii^—O4—Pr1—O6	−69.17 (8)
C2—C1—O7—Pr1^iii^	109.6 (2)	C5—O4—Pr1—O4^vii^	141.96 (10)
O9—C4—O8—Pr1^iv^	−69.1 (3)	Pr1^iii^—O4—Pr1—O4^vii^	−27.89 (15)
C3—C4—O8—Pr1^iv^	110.1 (2)	C5—O4—Pr1—O9^viii^	−102.32 (13)
O8—C4—O9—Pr1^v^	9.8 (3)	Pr1^iii^—O4—Pr1—O9^viii^	87.84 (9)
C3—C4—O9—Pr1^v^	−169.47 (15)	C5—O4—Pr1—O5	7.57 (12)
C1—O6—Pr1—O8^vi^	−51.5 (2)	Pr1^iii^—O4—Pr1—O5	−162.27 (11)
C1—O6—Pr1—O7^vii^	161.5 (2)	C5—O4—Pr1—O1	−85.22 (13)
C1—O6—Pr1—O4^vii^	−126.0 (2)	Pr1^iii^—O4—Pr1—O1	104.93 (9)
C1—O6—Pr1—O9^viii^	−115.5 (2)	C5—O4—Pr1—O1W	−31.27 (13)
C1—O6—Pr1—O5	73.8 (2)	Pr1^iii^—O4—Pr1—O1W	158.88 (7)

Symmetry codes:  (i) −*x*+1, −*y*, −*z*+1; (ii) −*x*+2, −*y*, −*z*+1; (iii) *x*, −*y*+1/2, *z*+1/2; (iv) *x*−1, *y*, *z*; (v) *x*−1, −*y*+1/2, *z*+1/2; (vi) *x*+1, *y*, *z*; (vii) *x*, −*y*+1/2, *z*−1/2; (viii) *x*+1, −*y*+1/2, *z*−1/2.

### Hydrogen-bond geometry (Å, °)

**Table:**

*D*—H···*A*	*D*—H	H···*A*	*D*···*A*	*D*—H···*A*
C3—H3···O7	0.93	2.50	2.815 (3)	100.
O1W—H2W···O7^ix^	0.83 (1)	2.11 (1)	2.911 (2)	165 (2)
O2—H2A···O9^viii^	0.82	1.86	2.661 (2)	167.
O1W—H1W···O5^x^	0.82 (1)	2.09 (2)	2.816 (2)	147 (2)

Symmetry codes:  (ix) −*x*+1, *y*−1/2, −*z*+1/2; (viii) *x*+1, −*y*+1/2, *z*−1/2; (x) −*x*+1, −*y*, −*z*.
